# Pregabalin for Neuropathic Pain in Post-radiotherapy Head and Neck Cancer Patients: A Retrospective Study and Review of the Literature

**DOI:** 10.7759/cureus.72951

**Published:** 2024-11-03

**Authors:** Maria Kouri, Erofili Papadopoulou, Emmanouil Vardas, Maria Georgaki, Martina Rekatsina, Athanasia Tsaroucha, Alberto Pasqualucci, Athina Vadalouca, Giustino Varrassi, Nikolaos G Nikitakis

**Affiliations:** 1 Anesthesiology, National and Kapodistrian University of Athens, Athens, GRC; 2 Oral Medicine and Pathology and Hospital Dentistry, School of Dentistry, National and Kapodistrian University of Athens, Athens, GRC; 3 Oral Medicine, National and Kapodistrian University of Athens, Athens, GRC; 4 Pain Management, Basildon University Hospital, London, GBR; 5 Anesthesiology and Critical Care, University of Perugia, Perugia, ITA; 6 Pain and Palliative Care Center, Athens University Hospital, Athens, GRC; 7 Pain Medicine, Fondazione Paolo Procacci, Rome, ITA

**Keywords:** analgesia, dn4 diagnostic questionnaires, neuropathic orofacial pain, nrs pain score, pregabalin

## Abstract

Introduction: Head and neck cancer (HNC) patients may experience neuropathic pain (NP) due to radiotherapy (RT), which may become chronic. Pregabalin, an anticonvulsant, alters the transmission of painful stimuli at the synaptic level, modifying their perception. Pregabalin is used in NP treatment, but limited data exist on RT-treated HNC patients. This retrospective study aimed to present a case series of HNC patients with chronic NP after RT managed with pregabalin.

Methods: HNC patients’ records from the Department of Oral Medicine and Pathology and Hospital Dentistry, School of Dentistry, National and Kapodistrian University of Athens (NKUA), were searched. Outcome measures were obtained using the Douleur Neuropathique 4 (DN4) scale for NP and the numeric rating scale (NRS) for pain. The use of additional analgesic medication was also recorded.

Results: Five HNC (four oral and one nasopharyngeal cancer) patients (mean age 58.5 years) who had received RT (mean total dose 64.7 gray (Gy)) and developed chronic (i.e., present for at least three months after RT) NP, characterized by a positive DN4 score ≥4, were identified. The initial assessment was five months to six years after RT (mean DN4=4.6±0.89 and mean NRS=6±3.08). “Burning,” “pins and needles,” and “numbness” were the NP descriptors mostly used. Pregabalin was titrated up to 150-300 mg per day; paracetamol and/or tramadol were also administered (daily doses 3000 mg and 100-150 mg, respectively). A substantial pain relief (≥50%) according to the Initiative on Methods, Measurement, and Pain Assessment in Clinical Trials (IMMPACT) (mean DN4=1.6±1.67 and NRS=1.6±1.67) was reported after two to three months of treatment, when tapering was initiated. Two patients (2/5) had complete remission of symptoms (DN4=0, NRS=0). No serious adverse effects were reported; one patient reported excess salivation.

Conclusions: Pregabalin may be a promising option for managing RT-related chronic NP in HNC patients. Further studies, including randomized controlled trials on RT-treated HNC patients, should be conducted.

## Introduction

Radiotherapy (RT) is part of the multimodal approach needed for the treatment of head and neck cancer (HNC) either as a stand-alone therapy or as a combination with surgery, chemotherapy, targeted therapy, and/or immunotherapy [1,2]. The therapeutic schema depends on the cancer location, histopathologic type and stage, and prior antineoplastic treatments; almost 75% of HNC patients receive RT [3,4]. Even though current treatments are extremely advanced and different RT techniques, such as intensity-modulated RT (IMRT), image-guided RT (IGRT), and volumetric modulated arc therapy (VMAT), have significant advantages, RT-related toxicity should be taken into consideration. There are still acute (developing during therapy) and chronic (developing months to years after therapy) complications, which may have a major impact on survival and quality of life (QoL) [5], as well as significant economic consequences [6].

RT-related oral pain is one of the most debilitating, prevalent, and feared consequences [7], resulting in increased use of opioid medications, need for hospitalization, use of feeding tubes, treatment breaks or cessation, and increased medical costs [8]. RT-related oral pain may be nociceptive or neuropathic; however, even when neuropathic pain (NP) is not the principal component of pain, one or more NP characteristics may be present. In our previously conducted study, 65.38% of HNC patients under RT reported at least one NP descriptor [7]. Approximately 20% of HNC patients with moderate or severe pain will develop NP during RT [9,10].

Acute RT pain usually develops during RT and may last up to one or two months after treatment. However, in some patients, pain may persist for years [7,11]. Data on chronic pain characteristics and prevalence in HNC patients after RT are limited, giving a prevalence of 15% to almost 60% of survivors [12,13]. In the study by Kallurkar et al. [13], most of the survivors reported severe pain using NP descriptors. Various treatments have been investigated for RT-related, acute pain [14-16]. However, chronic pain with a neuropathic component and chronic NP management have rarely been studied [11], leaving an unmet clinical need. NP is usually severe, persistent, and refractory to the usual analgesics, such as nonsteroidal anti-inflammatory drugs (NSAIDs), paracetamol, and opioids, used in nociceptive pain [17]. Moreover, NP has a negative impact on QoL, leading to a great burden for patients and a high demand for resources in the health care system [5].

Pregabalin is an anticonvulsant drug and a structural derivative of γ-aminobutyric acid (GABA) and is approved for NP treatment [18,19] as it alters the painful stimuli transmission mechanisms at the synaptic level, modifying their perception. Pregabalin binds to the neurons’ α2δ synaptic voltage-gated calcium channel (VGCC) subunits 1 and 2 [20], inhibiting the release of excitatory neurotransmitters as a result of calcium ion influx reduction [21]. Pregabalin is recommended for various NP conditions, such as painful diabetic neuropathy and postherpetic neuralgia [17], but there is a paucity of data on HNC patients treated with RT [11,22,23]. In one study by Jiang et al. [24] on the effect of pregabalin on post-RT pain in HNC patients, the authors reported a significant reduction in pain intensity in the pregabalin arm versus the placebo arm.

To the best of our knowledge, the aforementioned study [24] is the only one addressing the issue of post-RT-related NP in HNC patients after the administration of pregabalin, even if with several adverse events. Since there is a paucity of data in the literature, we conducted a retrospective search regarding pregabalin administration in HNC patients previously treated with RT in the files of the Dental Oncology Unit, Oral Medicine and Pathology and Hospital Dentistry, School of Dentistry, National and Kapodistrian University of Athens (NKUA). This retrospective study aimed to present a case series of patients with NP after RT managed with pregabalin. The preliminary results were partially presented during the Multinational Association of Supportive Care in Cancer/International Society of Oral Oncology Annual Meeting on Supportive Care in Cancer, Nara, Japan, June 22-24, 2023.

## Materials and methods

A single-center, retrospective study was carried out to assess the effects of pregabalin administration on NP in HNC patients after RT. Patients’ records from the Dental Oncology Unit, Department of Oral Medicine and Pathology and Hospital Dentistry, School of Dentistry, NKUA, from December 2018 to July 2022, were searched. This study was performed in accordance with the Declaration of Helsinki for studies involving human patients and was approved by the Committee of Ethics for Research of the School of Dentistry, NKUA (No 655 on 08.08.2024). Informed consent was obtained from the participants included in the study.

Inclusion criteria

Patients with HNC who were older than 18 years, previously treated with RT, assessed for NP, and first treated with pregabalin at least three months after RT completion were included in the study. The “at least three months interval” between RT completion and the first evaluation of the patients was set to be able to pass the acute complications’ duration window and characterize the pain as chronic [25]. Patients treated only with chemotherapy were excluded. Patients with prior administration of pregabalin or gabapentin were also excluded. 

Measurements

In patients who reported at least one NP word descriptor (such as burning, pins and needles, electric shocks, numbness, itching, tingling, painful cold, hypoesthesia to touch, or hypoesthesia to prick), the Greek language-validated 10-item Douleur Neuropathique 4 (DN4) questionnaire [26] was administered in two timepoints: timepoint of the first evaluation (t0, at least three months after the end of RT) and timepoint of the second evaluation (t1, when patients reported amelioration of pain severity or intensity and the tapering of pregabalin was initiated). The maximum DN4 possible score is 10 with a cutoff score of ≥4, which is considered the cutoff value for the diagnosis of NP. Using the cutoff value of ≥4, DN4 questionnaire showed 78% specificity, 93% sensitivity, 84% negative predictive value, and 90% positive predictive value in the detection of the neuropathic element of pain [26].

Nociceptive pain was also evaluated, at t0 and t1, using a 0-10 numeric rating scale (NRS). Scores <5 were considered as mild pain, 5-7 as moderate, and ≥8 as severe pain. The NRS is an established and validated instrument considered as a reliable pain intensity measure. Pain can be measured adequately by unidimensional tools [27]; when pain experienced in the previous 24 hours is assessed, NRS has a significantly higher descriptive capability than verbal rating scales in distinguishing between background and peak pain [28].

For the interpretation of the clinical importance of pain intensity (NRS assessment) outcomes, the Initiative on Methods, Measurement, and Pain Assessment in Clinical Trials (IMMPACT) [29] was used, defined as minimally important, at 10-20% pain relief; moderately important, at ≥30% pain relief; and substantial, at ≥50% pain relief.

Data collection

Data were collected using a Microsoft Excel sheet (Microsoft Corporation, Redmond, Washington). Demographic features such as age and gender, cancer location, and cancer therapy were collected. Data were also collected regarding the characteristics of pain, such as severity and nature of pain, and the use of pregabalin, opioids, and paracetamol. All data were evaluated and reported as mean values plus or minus standard deviation (mean (±SD)) and percentages (%).

Clinical parameters were assessed, including the time of pain at the first evaluation after RT, pain scores, NP descriptors, DN4 and NRS evaluation scores, management, and treatment outcomes after management.

## Results

From our repository, encompassing a total of 32 patients, five of them (mean age 58.5 years, range 47-71 years) who had received RT (mean total dose 64.7 gray (Gy)) for HNC were identified and included in this report. Four of them had oral cancer and one had nasopharyngeal cancer (Table 1).

**Table 1:** Demographics of included patients Gy, gray; RT, radiotherapy

Parameters		N=5	%
Gender	Male	3	60
	Female	2	40
Age (mean, years)	58.5		
Cancer site	Oral	4	80
	Nasopharyngeal	1	20
RT	Postoperative	4	80
	Stand-alone	1	20
Total RT dose (mean Gy)	64.7		
Concomitant chemotherapy		3	60

All patients were diagnosed with chronic NP in the oral cavity, with a positive DN4 score ≥4 (mean DN4=4.6±0.89), while the mean NRS was 6±3.08. Patients were first assessed five months up to six years after RT. “Burning,” “pins and needles,” “electric shocks,” and “numbness” were the NP descriptors mostly used (100%, 100%, 60%, and 80%, respectively) at t0 (Table 2).

**Table 2:** Neuropathic pain descriptors of each patient according to the DN4 questionnaire DN4, Douleur Neuropathique 4; NP, neuropathic pain; Pt, patient; t0, first evaluation; t1, second evaluation

NP descriptors	Pt 1 t0/t1	Pt 2 t0/t1	Pt 5 t0/t1	Pt 6 t0/t1	Pt 7 t0/t1	Total t0/t1
Burning	+/-	+/-	+/+	+/+	+/-	5/2
Pins and needles	+/-	+/-	+/+	+/+	+/-	5/2
Electric shocks	-/-	-/-	+/+	+/+	+/-	3/2
Numbness	+/-	-/-	+/+	+/+	+/+	4/3
Itching	-/-	+/-	+/+	-/-	-/-	2/1
Tingling	-/-	+/-	-/-	-/-	-/-	1/0
Painful cold	+/-	-/-	-/-	-/-	-/-	1/0
Hypoesthesia to touch	-/-	-/-	-/-	+/-	-/-	1/0
Hypoesthesia to prick	-/-	-/-	-/-	+/-	-/-	1/0
Brushing	-/-	-/-	-/-	-/-	-/-	0/0

For chronic NP, all patients received pregabalin, which was titrated to 150-300 mg per day (mean dose 245 mg), divided into one dose every 12 hours; paracetamol (daily dose 3000 mg) and/or tramadol (100-150 mg per day, mean daily dose 133.3 mg) were also administered (Table 3).

**Table 3:** Clinical pain-related parameters PG, pregabalin; RT, radiotherapy; NPG, nasopharyngeal; NRS, numeric rating scale; t0, first evaluation; t1, second evaluation

Patients	Cancer site	t0 (time after RT)	DN4 (t0)	NRS (t0)	PG (mg/day)	Other medication (mg/day)	t1 (time after PG initiation)	DN4 (t1)	NRS (t1)
1	NPG	3 years	4	10	300	Tramadol (150)	3 months	0	0
2	Oral	6 years	4	4	225	Paracetamol (3000)	3 months	0	0
3	Oral	6 months	5	7	300	Tramadol (100)	2 months	5	4
4	Oral	7 months	6	2	150	-	2 months	4	2
5	Oral	5 months	4	7	250	Tramadol (150) and/or paracetamol (3000)	3 months	1	2

Regarding pain relief outcomes (Table 3), according to IMMPACT [27], a reduction in mean DN4 and NRS scores (mean DN4=1.6±1.67 and NRS=1.6±1.67) was reported in two to three months of treatment duration, which was ≥50% pain relief and considered substantial. Two patients (40%) had complete remission of symptoms at t1 (DN4=0, NRS=0). There was also an improvement in NP descriptors use (Table 2).

No serious adverse effects were reported; excess salivation was reported in one patient. Three patients discontinued the drug completely. At one-year follow-up, there was no relapse of pain. One patient tapered the drug to 50 mg per day. She continued 50 mg per day for six months, after which the drug was discontinued, with DN4=1 and NRS=1-2. At one-year follow-up, there was no relapse. One patient tapered the drug to 25 mg. He did not come for follow-up (which may suggest that there was no pain).

## Discussion

The main aim of this study was to explore the efficacy of pregabalin in patients with NP after RT for HNC. All five patients included in the study had positive results and marginal side effects with the treatment.

The five-year relative survival rates for HNC have increased; in the United States, there is an improvement from 53% (years 1975-1977) to 69% (years 2013-2019) for oral cavity and pharyngeal cancers [30]. Thus, at the same time, there should be an increase in the prevalence of HNC post-RT survivors reporting chronic pain with or without neuropathic characteristics.

According to the WHO pain ladder modified by Vadalouca et al. [31], mild pain in cancer patients is treated with non-opioid medication (NSAIDs and/or paracetamol), while weak and strong opioids can be added for moderate and severe pain, respectively [32,33]. When NP exists, the addition of adjuvant analgesics such as pregabalin is mandatory since NP is usually refractory to the usual nociceptive analgesic medication [17].

Pregabalin alters the painful stimuli transmission mechanisms, modifying their perception, and is recommended in various NP conditions [17]. In the study by Jiang et al. [24] on post-RT pain in HNC patients, pain intensity score decreased in the pregabalin group by 2.44 points in NRS and 29.7% of patients achieved ≥50% pain relief. In our study, the NRS score decreased by 4.4 points and ≥50% pain relief was reported by 60% of patients (3/5). In this case report, patients 1 and 2 (2/5, 40%) had complete remission of symptoms at t1 (DN4=0, NRS=0). Patients 3 and 5 had intermediate (≥30%) and substantial (≥50%) pain relief, respectively, according to IMMPACT [29]. Patient 4 had no change in NRS scale scores; the patient reported mild pain and there was no need for administration of paracetamol or tramadol as nociceptive analgesics. In patients 3 and 4, the intensity of the NP descriptors reported was decreased despite the persistence of positive DN4 scores.

In our study, chronic NP was reported five months to six years after RT. In the review by Kuo and Williams [34], persistence of pain was reported up to 24 months after RT, while in the study by Taylor et al. [7] including 1114 HNC survivors five years or more after diagnosis, 14% of them reported pain. The neuropathic components of pain in these studies are not reported, although persistent unrelieved nociceptive pain along with RT-related neuronal injury can result in abnormal processing of noxious stimuli and therefore in NP [10,35].

The NP descriptors mostly used among our patients were “burning” (5/5), “pins and needles” (5/5), “numbness” (4/5), and “electric shocks” (3/5). In previous studies, burning, as an NP descriptor in HNC patients under RT, did not have any statistically significant difference between patients with positive and patients with negative scores in the NP evaluation, while “electric shocks” showed statistically significant differences [9,36]. This could indicate that the presence of RT-related NP in HNC patients may be suspected by clinicians upon reporting certain pain characteristics, such as “electric shocks.”

Tramadol’s analgesic properties derive from binding with the μ-opioid receptor and inhibiting the reuptake of norepinephrine and serotonin [37]. The oral mucosa’s peripheral sensory terminals present µ-opioid receptors [38]. The better analgesic effect provided by the combination of pregabalin and tramadol was reported by studies in humans [39] and animals [40]. No clinically significant drug interactions between pregabalin and tramadol were established when administered to healthy volunteers [41].

Pregabalin was well tolerated by the patients and no adverse events, such as dizziness and/or somnolence, were reported, due to slow titration. Only one patient (#2) reported excess salivation, right after the initiation of pregabalin administration, which in the RT-treated HNC patients’ population may not constitute an adverse event, since these patients most often suffer from xerostomia. The binding of pregabalin to the α2-δ subunit of calcium channels, which leads to decreased secretion of neurotransmitters, including norepinephrine [42], could explain the sialorrhea since norepinephrine targets the α2-adrenergic receptors that inhibit saliva secretion [43]. In other words, the depletion of norepinephrine induced by pregabalin could lead to excess salivation [43,44].

A limitation of this study is the retrospective design. Moreover, the small number of patients included and the heterogeneity in their clinical characteristics and oncologic treatment are a further limitation. In addition, the set timeframe of three months may have excluded patients who received pregabalin in the first three months and had a positive effect. Therefore, findings should be interpreted with caution and not generalized. Further studies, ideally prospective, randomized controlled trials with larger well-defined samples, should be conducted to investigate the effects of pregabalin on post-RT NP in HNC patients; the latter may represent a potentially significant treatment modality, as nociceptive pain therapy usually does not address NP [45].

## Conclusions

Pregabalin was well tolerated in the patients studied. Our results show that it could be a medication of choice for post-RT NP treatment in HNC patients. Further studies including randomized controlled trials with a large number of HNC patients treated with RT are in order.
